# 251 Impact of nurse-driven de-isolation process on isolation precautions duration at a Pediatric Hospital

**DOI:** 10.1017/ash.2026.10620

**Published:** 2026-06-23

**Authors:** Hyeyeon Goh, Nam-ju Lee, Pyoeng Gyun Choe, Mimi Lee

**Affiliations:** 1 Seoul National University Hospital; 2 Seoul National University Hospital, Seoul, Republic of Korea

## Abstract

**Background:** Healthcare environmental services (EVS) staff play a critical role in infection prevention and control (IPC) through environmental cleaning and disinfection. However, EVS staff are often excluded from structured, role-specific IPC education. Insufficient IPC knowledge among EVS staff may compromise environmental hygiene and increase the risk of healthcare associated infection transmission. This study aimed to assess IPC knowledge and education needs among EVS staff to provide an evidence-based foundation for targeted IPC education programs. Methods A descriptive cross-sectional study was conducted among EVS staff at a tertiary-care university hospital in Seoul, Republic of Korea. Participants completed a self-administered questionnaire assessing demographic characteristics, IPC knowledge, and perceived IPC education comprehension (current level) and education need (required level). IPC knowledge was measured using a 20-item instrument developed based on national IPC guidelines. Education needs were analyzed using the Borich’s needs assessment model and the Locus for Focus model to identify priority education topics. Descriptive statistics and nonparametric analyses were performed. Results A total of 81 EVS staff participated. The median IPC knowledge score was 17.0 (IQR, 16.0-18.0) out of 20. Notably, despite the high overall score, critical knowledge deficits were identified in high-risk IPC areas, including personal protective equipment (PPE) doffing protocols after cleaning an isolation room (42.0% correct) and management of infectious medical waste (17.3%). Education comprehension scores were lowest for management of suspected infection in staff, blood spill disinfection, and the safe use of disinfectants, whereas education need scores were highest for PPE related to various transmission-based precautions, as well as responses to needlestick injuries. Needs assessment using the Borich and Locus for Focus model identified procedures for managing suspected infection in staff (Borich score, 2.47), PPE for airborne precaution (2.00), and PPE for contact precaution (1.94) as the highest-priority IPC education topics, all of which were located in the high importance–high discrepancy (HH) quadrant; needlestick injury management and blood spill disinfection were also identified as high-priority areas. Conclusions Despite relatively high overall IPC knowledge, EVS staff demonstrated significant knowledge–practice gaps in high–risk IPC practices and expressed a substantial need for clearer guidelines essential for self-protection. The discrepancy between current understanding and perceived educational needs underscores the importance of structured, role–specific IPC education. Targeted IPC education programs focusing on isolation precautions, PPE donning and doffing, and occupational exposure management are crucial to enhancing both staff safety and the integrity of hospital-wide infection prevention.

